# The glycemic control has improved in Japanese patients with childhood-onset type 1 diabetes mellitus since 1995

**DOI:** 10.1186/1687-9856-2015-S1-O30

**Published:** 2015-04-28

**Authors:** Shin Amemiya, Mie Mochizuki, Toru Kikuchi, Tatsuhiko Urakami, Tomoyuki Kawamura, Nobuyuki Kikuchi, Nobuo Matsuura, Nozomu Sasaki, Shigetaka Sugihara

**Affiliations:** 1 Saitama Medical University, Saitama, Japan; 2 Yamanashi University, Yamanashi, Japan; 3 Nihon University, Tokyo, Japan; 4 Osaka City University, Osaka, Japan; 5 Minato Red-cross Hospital, Yokohama, Japan; 6 Seitoku University, Chiba, Japan; 7 Tokyo Women’s University Medical Center East, Tokyo, Japan; 8 The Japanese Study Group of Insulin Therapy for Childhood and Adolescent Diabetes, Japan

## Purpose

The effect of basal-bolus insulin therapy on better glycemic control was documented in the DCCT study. We aimed to clarify whether the introduction of insulin analogues gave the good quality of life using basal-bolus insulin therapy without hypoglycemia to the most of pediatric and adolescent patients with type 1 diabetes mellitus (T1DM).

## Methods

Glycemic control was compared between the 1st in 1995, 2nd in 2000 and 3rd in 2008 cohorts of childhood-onset T1DM in Japan, consisting of 566, 749 and 803 patients, respectively. We examined the data of HbA1c, frequency of moderately severe hypoglycemia, insulin regimen, body mass index (BMI) and the SD score each during July and October.

## Results

The median values of HbA1c (% of glycemic goal <7.5%) were significantly improved between the subsequent three cohorts; 9.02% (18.6%), 8.10% (30.35) and 7.60% (44.1%), in 1st, 2nd and 3rd cohorts, respectively. In addition patients with HbA1c more than 9% were significantly decreased in that order of cohorts. The frequency of moderately severe hypoglycemia was 9 times/ 4 months/ 100 patients in 3rd cohort. The frequencies of multiple daily insulin or pump therapy were 34.5%, 59.7% and 80.7%, in that order. The daily insulin dose was higher in 3rd cohort in comparison with 1st cohort. While the height SD score significantly increased in 3rd cohort, the BMI SD scores in 2nd and 3rd cohorts were significantly higher than that in 1st cohort.

## Conclusion

We conclude that the introduction of insulin analogues, especially using the long-acting analogues since 2003, resulted in better glycemic control with good quality of life with less moderately severe hypoglycemic episodes in comparison with DCCT study. On the other hand we may have to pay attention to overweight without dietary advice.

On behalf of The Japanese Study Group of Insulin Therapy for Childhood and Adolescent Diabetes.

